# Reliability of a telephone interview for the classification of headache disorders

**DOI:** 10.3389/fneur.2023.1238266

**Published:** 2023-08-25

**Authors:** Anselm Angermaier, Andy Koennecke, Christine Kloetzer, Sebastian Strauss, Robert Fleischmann

**Affiliations:** Department of Neurology, University Medicine Greifswald, Greifswald, Germany

**Keywords:** questionnaire, telephone interview, reliability, classification, agreement

## Abstract

**Objective:**

The study aimed to test the reliability of a semi-structured telephone interview for the classification of headache disorders according to the ICHD-3.

**Background:**

Questionnaire-based screening tools are often optimized for single primary headache diagnoses [e.g., migraine (MIG) and tension headache (TTH)] and therefore insufficiently represent the diagnostic precision of the ICHD-3, which limits epidemiological research of rare headache disorders. Brief semi-structured telephone interviews could be an effective alternative to improve classification.

**Methods:**

A patient population representative of different primary and secondary headache disorders (*n* = 60) was recruited from the outpatient clinic (HSA) of a tertiary care headache center. These patients completed an established population-based questionnaire for the classification of MIG, TTH, or trigeminal autonomic cephalalgia (TAC). In addition, they received a semi-structured telephone interview call from three blinded headache specialists individually. The agreement of diagnoses made either using the questionnaires or interviews with the HSA diagnoses was evaluated.

**Results:**

Of the 59 patients (*n* = 1 dropout), 24% had a second-order and 5% had a third-order headache disorder. The main diagnoses were as follows: frequent primary headaches with 61% MIG, 10% TAC, 9% TTH, and 5% rare primary and 16% secondary headaches. Second-order diagnosis was chronic migraine throughout, and third-order diagnoses were medication overuse headache and TTH. Agreement between main headaches from the HSA was significantly better for the telephone interview than for the questionnaire (questionnaire: κ = 0.330; interview: κ = 0.822; *p* < 0.001). Second-order diagnoses were not adequately captured by questionnaires, while there was a trend for good agreement with the telephone interview (κ = 0.433; *p* = 0.074). Headache frequency and psychiatric comorbidities were independent predictors of HSA and telephone interview agreement. Male sex, headache frequency, severity, and depressive disorders were independently predictive for agreement between the questionnaire and HSA. The telephone interview showed high sensitivity (≥71%) and specificity (≥92%) for all primary headache disorders, whereas the questionnaire was below 50% in either sensitivity or specificity.

**Conclusion:**

The semi-structured telephone interview appears to be a more reliable tool for accurate diagnosis of headache disorders than self-report questionnaires. This offers the potential to improve epidemiological headache research and care even in underserved areas.

## Introduction

Headache disorders are a great burden on the general population, resulting in reduced quality of life and job performance. There are effective treatment options which, however, have to be individualized on the basis of the correct diagnosis. However, making the correct diagnosis can be challenging for physicians not specialized in headache care as there are more than 200 distinct headache disorders defined by the international classification of headache disorders (ICHD-3) (1). Moreover, especially in rural areas, headache care must be maintained primarily by non-headache specialists (primarily primary care physicians) who are often not adequately trained (2, 3). Therefore, questionnaires have been developed to screen for main primary headache disorders such as migraine (MIG), tension-type headache (TTH), or trigeminal autonomic cephalalgias (TACs) for both clinical routine and research. Although a few headache questionnaires were validated for more than one disorder, these show poor performance in detection rate and accuracy of the diagnosis when a combination of different headache disorders is present (e.g., classification of MIG with trigeminal autonomic cephalalgia symptoms as TAC) (4). Many epidemiologic headache studies were conducted before the publication of the ICHD3 classification so that heterogeneous data exist, particularly in the prevalence of rare primary and secondary headaches (5, 6). Short semi-structured interviews *via* telephone might be an alternative option to improve detection rates. In this study, we investigated the reliability of a semi-structured telephone interview identifying different headache disorders in comparison to a questionnaire validated and used for epidemiological headache research (4) and our outpatient headache clinic (gold standard).

## Methods

**Figure 1:**
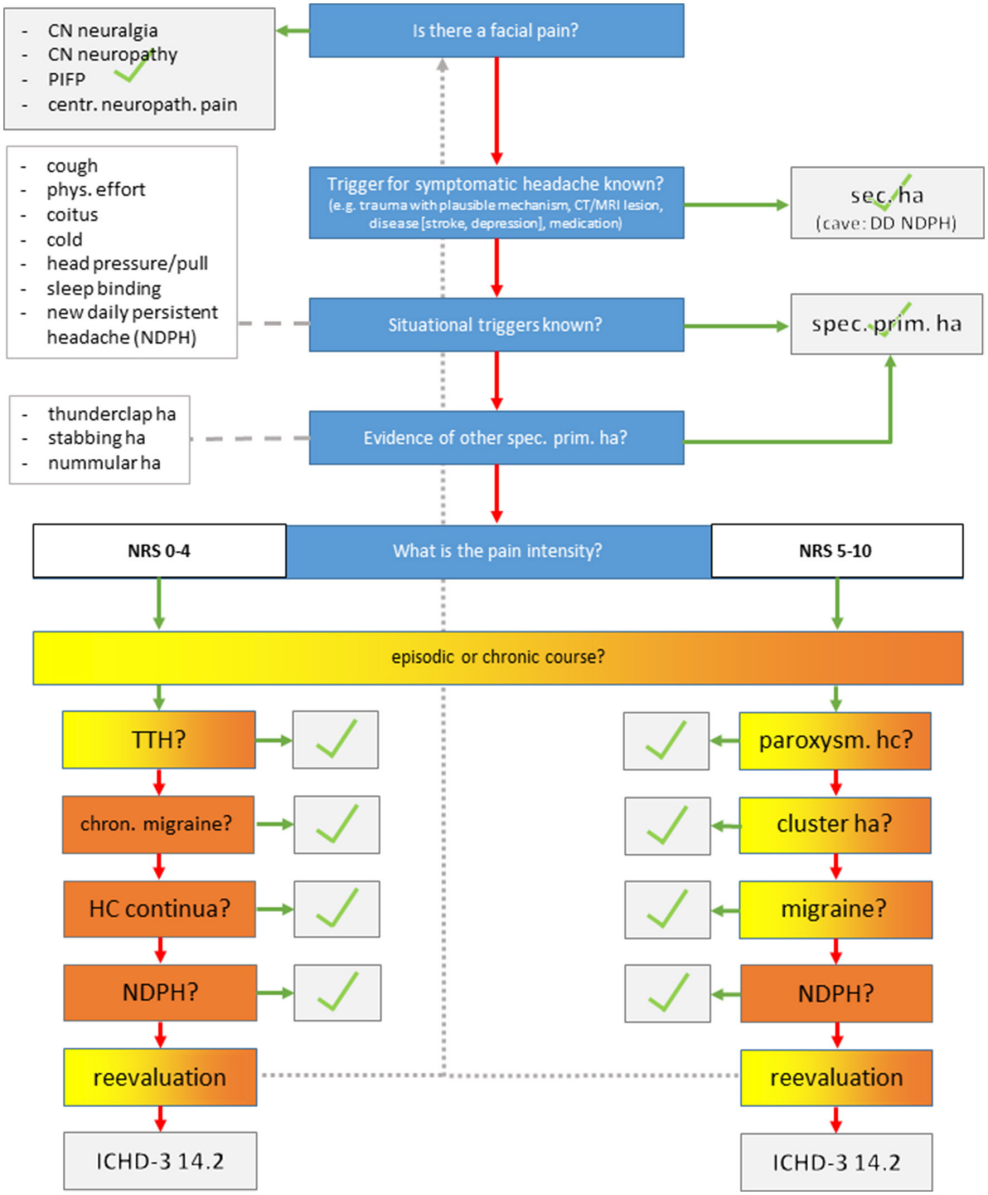
Semi-structured telephone interview. CN, cranial nerve; PIFP, persistent idiopathic facial pain; ha, headache.

The study was performed as a blinded observational study in our outpatient headache clinic and approved by the local ethics committee (BB 085/21). Known patients diagnosed with one or more headache disorders according to outpatient consultation and classified according to the ICHD-3 criteria were identified through a chart review. Care was taken to include both primary and secondary headaches and also frequent and rare headache disorders to keep the interviewers unaware of an a priori probability for certain diagnoses For this purpose, we screened the database starting with headache diagnoses that were least common and increasing to more common diagnoses (i.e., headache disorders were sorted by frequency in the database). We then contacted the identified patients and asked if they were willing to participate in the study. Since migraine is by far the most prevalent diagnosis, the remaining places according to the power analysis were filled with patients suffering episodic/chronic migraine with or without MOH, which yielded the final study sample. After inclusion, they prospectively completed a questionnaire that was validated and used for epidemiological headache research (4). This questionnaire was chosen because, to the best of our knowledge, there was no other questionnaire validated for the detection of more than one headache disorder in German and English language. Briefly, after explaining the principles and general rules for answering, the questionnaire continues with specific questions regarding MIG (seven items), TTH (seven items), and TAC (six items). The questions in the questionnaire were to be answered with “yes” or “no.” There are additional questions on the number of intake days of acute pain or migraine drugs per month. Questions and analysis algorithms are based on the classification criteria of the ICHD-2 (4, 7, 8). Questionnaires were sent to the patients' home addresses with an instruction to complete them and send them back using an envelope provided along with the letter. Later, they were called separately by three different headache specialists performing a semi-structured telephone interview for 10 min at the most (flow chart in Figure 1). The interview starts by exploring facial pain, secondary headache, and rare primary headache disorders, which are characterized by situational triggers and specific features. The interview then continues with pain intensity and frequency of headaches and specific phenomenological characteristics. Finally, a headache diagnosis was determined. Revaluation of the diagnostic interview was possible at any time in case of a new information provided by the patient. There were no predefined specific questions. In the case of several headache disorders, the diagnoses were sorted according to two criteria: (1) the amount of impairment caused by the disorder, which was generally the reason for consultation in the first place (i.e., migraine > TTH; TAC > TTH; if migraine + TAC co-exists, the one with more impairment was considered primarily). (2) in case of diagnoses that are not independent, causality was used to sort the data (i.e., you need a migraine to develop chronic migraine, and medication overuse headache is often a consequence of chronic migraine although disentanglement may be difficult if both co-exist for quite some time). In this case, migraine would be first-order, chronic migraine would be second-order, and MOH would be a third-order headache. Patients were instructed upon study inclusion beforehand to remain anonymous and neither to tell nor to provide hints regarding their headache diagnosis. The interview resulted in one or more headache disorders using the ICHD-3.

### Sample size considerations and statistics

Migraine is among the most frequent and bothersome headache disorders, and the sample size was thus adjusted to detect migraine patients among the sample population (9). Assuming an alpha error of 5% and a beta error of 80%, the McNemar test for paired observation of a headache disorder (i.e., outpatient clinic as gold standard vs. telephone interview) based on the expected 75% probability of detecting migraine patients in the outpatient clinic revealed a sample size of 60 to detect at least 20% discrepancies in diagnosis, which would yield a non-superiority to the questionnaire.

Cohen's kappa was used for agreement between the outpatient headache clinic (gold standard) and the questionnaire/telephone interview; differences between raters in the telephone interview were analyzed by the chi-squared test; stepwise binary logistic regression analysis was used for identifying predictors for agreement between gold standard and questionnaire or telephone interview. A predictor was kept in the model if the *p*-value was lower or equal to 0.157, which is the cutoff for an optimization of the model based on the Akaike information criterion (10). There were no missing values or outliers. Only patients with complete outpatient clinic data were selected.

## Results

Fifty-nine patients were recruited between 1 March and 4 April 2022, and one patient dropped out (did not respond to phone calls). Telephone interviews were performed within 2 weeks after inclusion. All patients were interviewed by the headache specialists. The majority of patients were women (68%), and the median age was 50 years [interquartile range (IQR) 33–39]. Median headache frequency was 8 days per month (IQR 4–13), and patients were moderately to severely affected in the Headache Impact Test-6 (HIT-6) (median: 60; IQR 53– 64) and Migraine Disability Assessment (MIDAS) (median: 29; IQR: 10–49). A total of 24% of patients suffered more than one disorder and 5% suffered a third-order headache disorder (Table 1).

**Table 1:** Baseline characteristics.

	**N (%)**	**Median**	**Mean**	**IQR**
Age		50	47	33–59
Headache frequency (days per month) other than TAC^*^	53 (90)	8	11	4–14
Headache frequency (days per month) Only TAC	6 (10)	3.5	7.5	0–8
MIDAS^**^		29	33	10–49
HIT-6^***^		60	57	53–64
Sex (female)	40 (68)			
Depression	18 (31)			
Psychological comorbidity	24 (41)			
Chronic pain syndrome	5 (8)			
One headache disorder	59 (100)			
Two headache disorders	14 (24)			
Three headache disorders	3 (5)			

* TAC, trigeminal autonomic cephalalgia;

** MIDAS, Migraine Disability Assessment Score;

*** HIT-6, Headache Impact Test-6.

The frequency of first-order headache diagnoses was 61% MIG, 10% TAC, and 9% TTH; others were rare primary (5%) or secondary headaches (16%). The second-order disorder was chronic migraine (*n* = 14), and all third-order disorders were medication overuse headache (*n* = 2) and TTH (*n* = 1). Agreement with first-order headache disorders from the headache outpatient clinic (HSA) was significantly better for the telephone interview than for the questionnaire [questionnaire: κ = 0.330; interview: κ = 0.822; *p* < 0.001)]. The second-order headache diagnosis was not adequately captured by questionnaires, while there was a trend for good agreement with a telephone interview (κ = 0.433; *p* = 0.074). There was no agreement in the third-order diagnosis either in questionnaires or telephone interviews.

MIG, TTH, and TAC showed moderate-to-fair agreement between questionnaires and HSA (kappa < 0,57; *p* = 0,037). There was no agreement in all other headache disorders. However, there was substantial-to-almost perfect agreement between the telephone interview and HSA in all first-order headache diagnoses (kappa ≥ 0,66; *p* < 0,001) (Table 2). There were no significant differences between the three telephone raters in determining first-order headache diagnosis (*p* = 0.72).

**Table 2:** Diagnosis-specific agreement HSA^†^ vs. questionnaire/telephone interview in the first diagnosis.

**Diagnosis HSA**^**†**^ **(ICHD-3) N (%)**		**Questionnaire**			**Telephone interview**		
		κ	**p**	**95 % CI**	κ	**p**	**95 % CI**
1 Migraine	36 (61)	0.57	<0.001	[0.29;0.85]	0.88	<0.001	[0.71;1.05]
2 TTH^‡^	5 (8)	0.43	0.002	[0.16;0.71]	0.69	<0.001	[0.52;0.86]
3 TAC^δ^	6 (10)	0.29	0.037	[0.02;0.57]	0.88	<0.001	[0.71;1.05]
4 Other primary ha^*^	3 (5)		ns		0.69	<0.001	[0.52;0.87]
5 Post-traumatic ha^*^	3 (5)		ns		1.00	<0.001	[0.83;1.18]
6 ha^*^ due to vascular disorder)	1 (2)		ns		0.74	<0.001	[0.57;0.92]
7 ha^*^ due to intracranial disorder	1 (2)		ns		0.66	<0.001	[0.49;0.84]
13 trigeminal neuropathy/facial pain	4 (7)		ns		0.85	<0.001	[0.67;1.02]

† HSA, outpatient headache clinic;

‡ TTH, tension-type headache;

δ TAC, trigeminal autonomic cephalalgia;

* ha, headache.

The agreement between HSA and the questionnaire was independently influenced by male sex, headache frequency, headache intensity, and depressive disorders (Table 3), whereas agreement with the telephone interview was only influenced by headache frequency and psychiatric comorbidity (Table 4). Looking at the performance scores, it is evident that the telephone interview performs significantly better than the questionnaire in detecting primary headache with high sensitivity (>88%) and specificity (>92%), especially in rare primary headache syndromes that are virtually impossible to detect with the questionnaire. The questionnaire has a moderate positive and negative predictive value for MIG and a high negative predictive value for TTH and TAC (Table 5). Performance scores for all headache disorders in our cohort can be found in Appendix.

**Table 3:** Stepwise backward multivariate analysis of factors influencing agreement between HSA^ψ^ diagnosis and questionnaire.

	**Starting model**			**Final model**		
	**OR** ^†^	**P**	**95% CI**	**OR** ^‡^	**p**	**95% CI**
MIG ^ 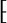 ^	0.33	ns^*^	0.25–4.34			
TTH^Ω^	0.11	ns	0.01–2.73			
TAC^δ^	0.07	ns	0.02–2.66			
Male sex	11.6	0.11	0.56–234.51	4.23	0.001	4.23–356.42
age	0.98	ns	0.91–1.06			
ha^||^ frequency	0.87	0.09	0.73–1.02	0.83	0.006	0.73–0.95
HIT-6^Δ^	0.97	ns	0.86–1.09			
MIDAS^Ψ^	1.05	0.08	0.99–1.11	1.05	0.02	1.01–1.09
Depressive mood	48.5	0.07	0.73–3213.54	1.19	0.04	1.19–1017.82
Chronic pain disorder	3.01	ns	0.02 – 382.03			
Psychological comorbidity	0.07	ns	0.03 – 1.69	0.12	ns	0.11 – 1.34
Constant	6.7	0.66		0.14	0.08	

* Not significant; stepwise binary logistic regression: OR > 1 increased likelihood of agreement; OR < 1 increased likelihood of no agreement;

† after step 1;

‡ after step 5;

ψ HSA, outpatient headache clinic;

^
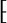
^MIG, migraine;

Ω TTH, tension-type headache;

δ TAC, trigeminal autonomic cephalalgia;

|| ha, headache;

Δ HIT-6, Headache Impact Test;

Ψ MIDAS, Migraine Disability Assessment Score.

**Table 4:** Stepwise backward multivariate analysis of factors influencing agreement between HSA^ψ^ diagnosis and telephone interview.

	**Starting model**			**Final model**		
	**OR** ^†^	**P**	**95% CI**	**OR** ^‡^	**P**	**95% CI**
MIG ^ 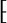 ^	0.35	ns	0.02–5.52			
TTH^Ω^	0.12	ns	0.01–1.62			
TAC^δ^	0.69	ns	0.02–23.49			
Male sex	0.71	ns	0.1–4.94			
Age	0.97	ns	0.91–1.03			
ha^||^ frequency	0.88	0.04	0.78–0.99	0.89	0.006	0.81–0.97
HIT-6^Δ^	1.05	ns	0.96–1.14	1.05	ns	0.99–1.11
MIDAS^Ψ^	0.99	ns	0.96–1.03			
Depressive mood	18.26	ns	0.81–409.52	8.33	ns	0.8–86.432
Chronic pain disorder	2.58	ns	0.04–167.17			
Psychological comorbidity	0.09	0.08	0.01–1.36	0.15	0.03	0.26–0.82
Constant	50.63	0.22		2.79	0.44	

* not significant; stepwise binary logistic regression: OR > 1 increased likelihood of agreement; OR < 1 increased likelihood of no agreement;

† after step 1;

‡ after step 6;

ψ HSA, outpatient headache clinic;

^
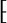
^MIG, migraine;

Ω TTH, tension-type headache;

δ TAC, trigeminal autonomic cephalalgia;

|| ha, headache;

Δ HIT-6, Headache Impact Test;

Ψ MIDAS, Migraine Disability Assessment Score.

**Table 5:** Performance measurements of telephone interview and questionnaire vs. HSA^ψ^ diagnosis (gold standard) for primary headache disorders.

		**MIG ^ 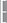 ^**	**TTH^Ω^**	**TAC^δ^**	**Other prim. HA^||^**
Telephone interview	Sens	92,68	83,33	88,89	71,43
	Spec	97,73	92,11	99,15	96,64
	PPV	98,70	52,63	88,89	55,56
	NPV	87,76	98,13	99,15	98,29
	LLR+	40,78	10,56	104,00	21,25
	LLR-	0,07	0,18	0,11	0,30
Questionnaire	Sens	93,33	100,00	33,33	0,00
	Spec	42,86	46,34	95,12	100,00
	PPV	77,78	12,00	33,33	na
	NPV	75,00	100,00	95,12	95,45
	LLR+	1,63	1,86	6,83	na
	LLR–	0,16	0,00	0,70	1,00

ψ HSA, outpatient headache clinic;

^
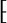
^MIG, migraine;

Ω TTH, tension-type headache;

δ TAC, trigeminal autonomic cephalalgia;

|| ha, headache; Sens, sensitivity; Spec, specificity; PPV, positive predictive value; NPV, negative predictive value; LLR+, positive likelihood ratio; LLR, negative likelihood ratio; na, not available (division by zero).

## Discussion

The results of our study showed that the semi-structured telephone interview performed more reliably in the classification of headache disorders than the self-reporting questionnaire. In addition, the results showed that there was a high general agreement on the clinical diagnosis of a headache clinic, making the interview an effective and valid screening tool.

The diagnosis-specific agreement between HSA and the questionnaire at the first headache diagnosis in our study was comparable to results from the validation study (4). The agreement was the best in the diagnosis of migraine, followed by TTH and TAC. Analogous to our study, agreement decreased in the presence of multiple headache diagnoses when the questionnaire was used (4, 8). Compared with a specific headache diagnosis questionnaire such as the Migraine ID, it results in lower agreement and lower sensitivity (11–13). Consequently, when using a headache diagnosis-specific questionnaire, the a priori test probability must be high which needs some diagnostic headache skills in the first place. The telephone interview showed significantly better agreement than the questionnaire in the diagnosis of the first headache entity, especially for MIG, TTH, and TAC, and opens the possibility to identify rare primary headache disorders. Consequently, performance scores for the telephone interview are significantly better.

To the best of our knowledge, this is the first study of a physician-based semi-structured telephone interview for the diagnosis of headache disorders. Generally, telephone interviews have been used more frequently in headache care (14) and also in primary headache disorder classification (15). Potter et al. developed a semi-structured telephone interview aimed to exclude rare primary and secondary headaches and differentiate between chronic TTH and MIG as well as medication overuse headache (MOH), which was conducted by untrained nurses (16). It was not a diagnostic interview as patients not having chronic TTH and MIG were sent to their GP for further evaluation. The overall agreement between headache specialists and nurses was only moderate. The German Robert Koch Institute designed a structured telephone interview detecting TTH, MIG, and MOH using the ICHD-3 criteria, which was applied in a German nationwide survey of 5,000 subjects done by lay personnel (17). Our semi-structured interview combines the advantages of the structured interview—structure allows a time-effective classification of the headache disorder—with the advantages of an unstructured interview—flexible response to the patient's answers. This examiner-dependent variability opens at the same time as the possibility of bias errors, which are not present in the self-report questionnaire (18, 19). Possible biases are the emergence of question order, context effects, the emergence of response-order effects, the validity of retrospective reports, and socially desirable responses (20). We do not believe that these effects are crucial here; after all, there was no significant difference between investigators in the first-order diagnosis. Designing self-reporting questionnaires is based on the operationalization of the ICHD criteria into layperson-understandable questions and on the use of possible filter questions. The depth of the desired classification has a significant influence on the scope of the questions to be asked and the complexity of the questionnaire (21). Coi et al. identified 48 possible causes of bias in designing and administering a questionnaire (22). Furthermore, they were prone to subjective assessment and thus subject to individual influencing factors. Thus, we identified several independent factors affecting agreement between the questionnaire and HSA diagnosis in our study. Male sex and more severe depressive symptoms were clearly associated with increased odds for an agreement of diagnoses. On the contrary, higher headache frequency led to an inferior agreement, which might be explained by tension-type phenotypes in patients with chronic migraine and/or MOH.

The telephone interview done by headache specialists offers the advantage of the entity-independent recording of headache disorders by an interactive review of ICHD3 criteria. In our study, agreement between the telephone interview and gold standard was much less confounded by headache characteristics and comorbidities than the questionnaire, rendering its results more robust. In addition, this approach offers the possibility of recording rare headache entities for which no validated questionnaires are available. Another advantage is the easy access that might allow large-scale use in underserved regions. For these reasons, it is of interest for use in epidemiological studies, especially to clarify how common rare headache syndromes really are. For routine clinical practice, the disadvantage of the need for limited available headache specialists to perform the interview in rural areas can be compensated by telemedicine approaches (23, 24). However, questionnaires are still an important tool in primary care, and more efforts are needed to be made to impart knowledge and skills about administering and interpreting the results in our study—moderate positive and negative predictive values for MIG and high negative predictive values for TTH and TAC.

This study has some limitations, which need to be addressed. It is a monocentric study, i.e., despite blinding, it was possible in individual cases that patients named their diagnoses or that the patient was known to the investigator by voice. Furthermore, a significant proportion of patients were affected by migraine rendering its detection more likely. However, investigators were unaware of the distribution of diagnoses, and secondary and rare primary headache disorders were equally well identified, which contradicts a selection bias in this study. Nonetheless, validation of results in an independent cohort is desirable.

## Conclusion

The semi-structured telephone interview appears to be a more reliable and accurate tool for the classification of headache disorders than self-report questionnaires. Main headache diagnoses were comparable to personal consultations in this study, a finding that requires confirmation in different settings. Nonetheless, our findings offer future potential to improve headache care even in previously underserved areas.

## Data availability statement

The raw data supporting the conclusions of this article will be made available by the authors, without undue reservation.

## Ethics statement

The studies involving humans were approved by Ethikkommission an der Universitätsmedizin Greifswald Institut für Pharmakologie Felix-Hausdorff-Str. 3 17487 Greifswald, Germany. The studies were conducted in accordance with the local legislation and institutional requirements. The participants provided their written informed consent to participate in this study.

## Author contributions

AA, AK, CK, SS, and RF contributed to conception and design of the study. AK included the patients and organized the database. AA, SS, and RF performed the telephone interview. RF performed the statistical analysis. AA wrote the first draft of the manuscript. All authors contributed to manuscript revision, read, and approved the submitted version.

## Appendix

**Table A1:** Performance measurements of telephone interview and questionnaire vs. HSA^ψ^ diagnosis (gold standard).

	**ICHD-3 Cat**	**MIG ^^**	**TTH^Ω^**	**TAC^δ^**	**Other primary HA^||^**	**5**	**6**	**7**	**13**
Telephone interview	Sens	92,68	83,33	88,89	71,43	100,00	100,00	100,00	75,00
	Spec	97,73	92,11	99,15	96,64	99,17	98,37	99,20	100,00
	PPV	98,70	52,63	88,89	55,56	83,33	60,00	50,00	100,00
	NPV	87,76	98,13	99,15	98,29	100,00	100,00	100,00	98,33
	LLR+	40,78	10,56	104,00	21,25	121,00	61,50	125,00	inf
	LLR-	0,07	0,18	0,11	0,30	0,00	0,00	0,00	0,25
Questionnaire	Sens	93,33	100,00	33,33	0,00	0,00	na	0,00	0,00
	Spec	42,86	46,34	95,12	100,00	100,00	100,00	100,00	100,00
	PPV	77,78	12,00	33,33	na	na	na	na	na
	NPV	75,00	100,00	95,12	95,45	95,45	100,00	97,73	93,18
	LLR+	1,63	1,86	6,83	na	na	na	na	na
	LLR-	0,16	0,00	0,70	1,00	1,00	na	1,00	1,00

ψ HSA, outpatient headache clinic;

^^MIG, migraine;

Ω TTH, tension-type headache;

δ TAC, trigeminal autonomic cephalalgia;

|| ha, headache; Sens, sensitivity; Spec, specificity; PPV, positive predictive value; NPV, negative predictive value; LLR+, positive likelihood ratio; LLR, negative likelihood ratio; inf, infinity; na, not available (division by zero).
